# Molecular Cross-Talk between Gravity- and Light-Sensing Mechanisms in *Euglena gracilis*

**DOI:** 10.3390/ijms23052776

**Published:** 2022-03-03

**Authors:** Adeel Nasir, Peter Rolf Richter, Aude Le Bail, Viktor Daiker, Julia Stoltze, Binod Prasad, Sebastian Michael Strauch, Michael Lebert

**Affiliations:** 1 Gravitational Biology Group, Department of Biology, Friedrich-Alexander University, Staudtstraße 5, 91058 Erlangen, Germany; adeel.nasir@fau.de (A.N.); aude.lebail@fau.de (A.L.B.); viktor.daiker@fau.de (V.D.); juliastoltze@web.de (J.S.); bin.aviansh@gmail.com (B.P.); michael.lebert@fau.de (M.L.); 2 Postgraduate Program in Health and Environment, University of Joinville Region, Rua Paulo Malschitzki, 10—Zona Industrial Norte, Joinville 89219-710, SC, Brazil; sebastian.michael@univille.br

**Keywords:** *Euglena gracilis*, phototaxis, gravitaxis, photoactivated adenylyl cyclase, protein kinase A, RNA interference, indirect immuno-fluorescent assay, movement analysis

## Abstract

*Euglena gracilis* is a photosynthetic flagellate. To acquire a suitable position in its surrounding aquatic environment, it exploits light and gravity primarily as environmental cues. Several physiological studies have indicated a fine-tuned relationship between gravity sensing (gravitaxis) and light sensing in *E. gracilis*. However, the underlying molecular mechanism is largely unknown. The photoreceptor photoactivated adenylyl cyclase (PAC) has been studied for over a decade. Nevertheless, no direct/indirect interaction partner (upstream/downstream) has been reported for PAC. It has been shown that a specific protein, kinase A (PKA), showed to be involved in phototaxis and gravitaxis. The current study reports the localization of the specific PKA and its relationship with PAC.

## 1. Introduction

Light and gravity are the most important environmental cues that many living organisms utilize to orient themselves in their surroundings [1,2,3,4,5,6]. Plants present a prominent example, as they perform directional movements in response to light (phototropism) and gravity (gravitropism) to orient themselves to a favorable niche for their development and growth. A growing body of evidence suggests that a synergistic effect of phototropism and gravitropism facilitates plant growth [1]. The involvement of auxin in phototropism and gravitropism further strengthens this phenomenon at the molecular level [7]. Similarly, free-swimming eukaryotic unicellulars are also largely dependent on light and gravity for their growth and survival [4,8]. Dissimilar to plants, there is not much known about the plausible underlying molecular mechanism that controls both light- or gravity-sensing responses in unicellular organisms. This study aims to understand the underlying molecular mechanism of interdependent responses of *Euglena gracilis* to light and gravity stimuli.

The photosynthetic unicellular flagellate, *E. gracilis*, belongs to a group of Euglenozoa and shares genetic homology with some notable parasites of the genera Trypanosoma and Leishmania [9,10]. *E. gracilis* contains chloroplasts that are surrounded by three membranes acquired by the secondary endosymbiosis of a green alga, even though Euglenozoa evolved independently from the Archaeplastida and green algae [11]. *E. gracilis* detects a range of environmental cues, such as oxygen, light, and gravity [4,8,12]. Interestingly, several physiological studies established that *E. gracilis* exploits gravity and light as significant environmental stimuli to reach an appropriate point in a water column for its survival [4,8,13].

Three types of photo-responses have been reported in *E. gracilis*, namely, photokinesis (light intensity-dependent changes in the linear swimming velocity) [14,15], phototaxis (directional swimming towards or away from light) [16], and photophobic reactions (a tumbling, transient freezing, or swimming backward upon a light intensity increase or decrease termed as step-up and step-down photophobic responses, respectively) [17]. The photoreceptor molecule, photoactivated adenylyl cyclase (PAC), is involved in step-up photophobic responses and phototaxis [17,18]. It is believed that PAC undergoes cyclase activity and produces cyclic adenosine monophosphate (cAMP) in blue light. This cAMP is believed to control the beating of flagella in *E. gracilis* [17,18]. The specific effector molecules, such as the protein kinase A (PKA), and the regulatory molecules, such as phosphodiesterase (PDA), of this blue light-dependent cAMP cascade are yet to be discovered.

Dissimilar to the light-sensing mechanism, *E. gracilis* exhibits only one type of gravity-mediated response: gravitaxis—directional swimming towards (positive gravitaxis) and away from gravity (negative gravitaxis) [19]. Primarily cells exhibit negative gravitaxis in natural habitats. However, under laboratory domestication conditions, cells show a clear transition of positive to negative gravitaxis as the culture grows from the exponential to stationary phase, respectively [19]. Furthermore, it has been demonstrated that several stressors also lead to the sign change (positive to negative and vice versa) in gravitaxis [20]. The underlying molecular mechanism of gravitaxis has not yet been exclusively deciphered. However, a current working model of gravitaxis is proposed based on a range of physiological, pharmacological inhibitor, and molecular biology experiments [4]. According to the model, the activation of stretch-sensitive calcium ion channels (SSCIC) leads to a calcium influx, following the pressure exerted by the upper cell membrane on the lower membrane, as *Euglena* deviates from its vertical swimming pathway. Subsequently, the calcium ions bind to a specific calmodulin which binds to adenylyl cyclase. The adenylyl cyclase activity leads to cAMP production, which activates a specific PKA. Subsequently, PKA modulates the flagellar movement through the phosphorylation of flagellar proteins. Until now, a transient receptor potential-like protein (TRP), PKA, calmodulin 2 (CaM2), and its interaction partner *E. gracilis* protein containing the domain of unknown function 4201 (EgPCDUF4201), have been identified as members of the gravitactic signal transduction chain using the RNA interference approach [21,22,23,24].

Evidence suggests that light- and gravity-sensing mechanisms overlap in *E. gracilis*. For instance, it has been demonstrated that the counterbalance between positive phototaxis and negative gravitaxis facilitates *E. gracilis* to orient itself in a water column [25]. However, the molecular basis of this overlapping mechanism is not explicitly clear yet. In this regard, the cAMP involvement and the PKA role in phototaxis and gravitaxis are of critical importance [17,21,26]. Therefore, a further investigation of the PKA is essential to understand its dual role in controlling gravity- and light-sensing mechanisms. This study reports the subcellular localization of PKA and its association with the photoreceptor PAC, which has been shown to regulate the cAMP pathway in light-sensing mechanisms in *E. gracilis* [17].

## 2. Results

### 2.1. PKA Resides in the Anterior Region of E. gracilis

The subcellular localization of PKA in *E. gracilis* was carried out using a genomic antibody. The anti-PKA antibody was generated using the genomic antibody (GAB) approach [27], which ensures the detection of an antigen in its native and denatured state. The anti-GAB-PKA’s reactivity and specificity were confirmed before the protein localization study. The expected size of a single band was obtained for various concentrations of *E. gracilis* protein lysate on an immunoblot treated with anti-PKA-GAB (Supplementary Figure S1a). To further determine the specificity of the antibody, the amount of PKA in the protein lysate of wild-type and RNAi-mediated PKA-silenced cells was analyzed. The PKA fraction was significantly lower in the protein lysate of PKA-silenced cells compared to the wild-type (Supplementary Figure S1b). These results corroborated the reactivity as well as the specificity of the anti-PKA-GAB.

**Figure 1:**
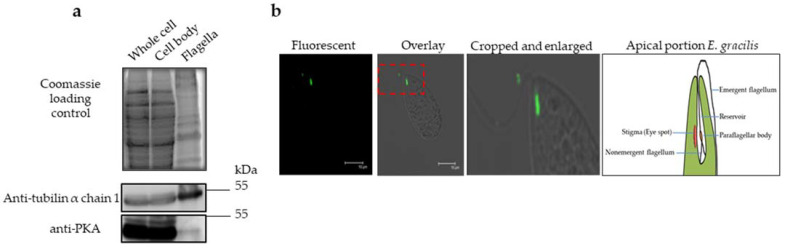
PKA localization in *E. gracilis*. (**a**) Western blots of the cell fractions. Coomassie loading control (upper panel), Western blot with tubulin antibody (middle panel), and PKA antibody (lower panel). (**b**) Fluorescent, overlay, and enlarged portion of the overlay image indicated by the dashed red rectangle as determined by the IIFA using the Anti-PKA antibody. Scale bar = 10 µm. The right sketch image shows the major organelles in the apical portion of *E. gracilis*.

The subcellular localization of PKA was performed by adapting an indirect immunofluorescence assay (IIFA) [28] and Western blot assay [23], followed by a cell fractionation assay. Cell fractionation was carried out using the calcium-shock method [29], resulting in the separation of the cell body and flagella. The total protein lysate for a fraction of an intact cell, isolated cell body, and flagella was compared for the amount of PKA on an immunoblot (Figure 1a). The PKA content was abundant in the cell body compared to the flagella fraction (Figure 1b). The IIFA showed that the PKA signal was confined all over the reservoir till its opening at the anterior end of the cell (Figure 1b). The decreased fluorescence in the PKA-silenced cells ensured the specificity of the signal (Supplementary Figure S2).

In addition to its role in nutrient uptake, the reservoir region contains a motor apparatus [30]. The motor apparatus consists of a short (nonemergent) flagellum and a long (emergent) flagellum. The long flagellum bears the paraflagellar body (PFB) at its base, harboring the photoreceptor PAC [17]. The PKA signal was diffused as determined by IIFA. Its precise association with organelles and the membrane of the reservoir region was challenging to determine. However, the recombinant PKA was collected from the soluble protein fraction (Supplementary Figure S1c). Therefore, it is implausible that PKA is associated with the membrane of the reservoir region. However, the organelle fractionation study and the IIFA suggested that PKA is associated with flagella and PFB. Moreover, previous studies have shown that PKA is involved in both light- and gravity-sensing mechanisms, while PAC exclusively controls the light-sensing mechanism in *E. gracilis* [17,18]. The proximity of PKA with PAC and their established roles strongly suggest a cross-talk in light- and gravity-sensing signal transduction.

### 2.2. Interdependency of PKA and PAC Expression Levels

**Figure 2:**
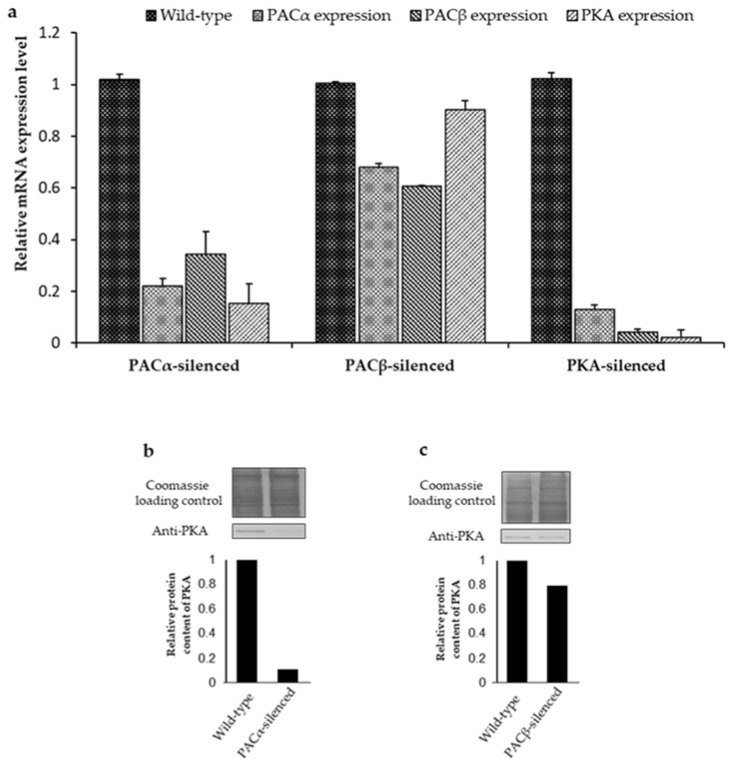
Coregulation of PKA, PACα, and PACβ expression levels. (**a**) Expression levels of PACα, PACβ, and PKA in PACα-, PACβ-, and PKA-silenced *E. gracilis* cells normalized to actin. (**b**,**c**) The protein level of PKA in PACα- and PACβ-silenced cells. All the transcript levels were normalized with the actin transcript level. These experiments were performed independently at least three times to ensure the reproducibility of the data.

**Figure 3:**
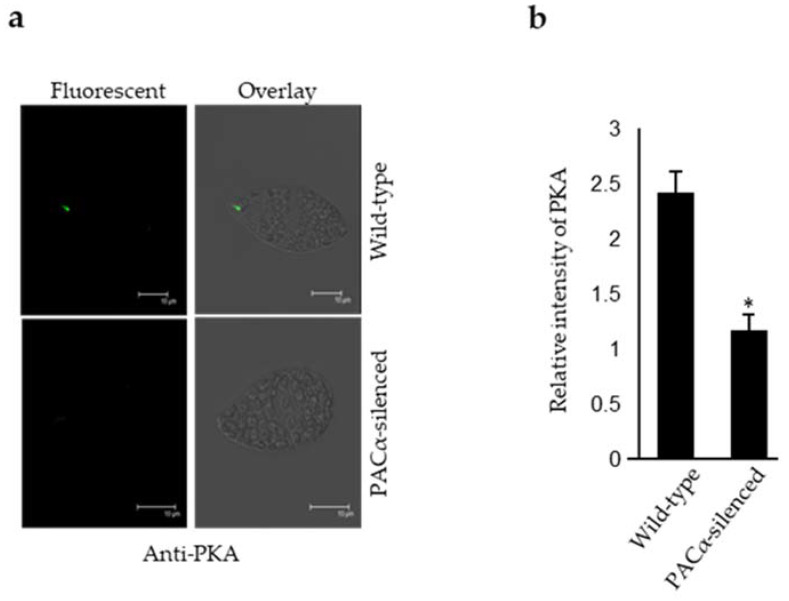
PKA content in PACα-silenced cells. (**a**) Fluorescent and overlay images of wild-type and PACα-silenced cells as determined by the IIFA using the Anti-PKA antibody. (**b**) Relative fluorescent intensity of PKA in wild-type and PACα-silenced cells. Error bars indicate standard error mean. *n* = 50 cells; * indicates a Student’s *t*-test *p*-value < 0.01.

It has been reported that members of the same signal transduction chain may co-regulate their expression level (mRNA/ protein) [31]. Therefore, PKA and PAC expression levels were determined in the PAC and PKA-silenced cells, respectively. The PAC consists of two α and β subunits, making it a tetrameric protein [17]. The N-terminal of both α and β subunits shares an overall homology, whereas their C-terminal is nonhomologous [17]. Moreover, PKA does not share any homology with both subunits of the PAC. The nonhomologous regions of α and β subunits of PAC were targeted to generate respective silenced cells. The expression level of PKA (mRNA/protein) and PACβ (mRNA) was found to be significantly lower in the PACα-silenced cells (Figure 2a,c). In PACβ-silenced cells, the expression level of PACα was highly reduced, whereas the PKA remained unaffected (Figure 2a,d). However, the silencing of PKA resulted in a reduction in both PACα and PACβ mRNA expression levels (Figure 2a). Furthermore, the decrease in the PKA content was confirmed in PACα-silenced cells via an IIFA (Figure 3a). Taken together, it was concluded that an interdependency of the expression level exists between PKA and PAC.

### 2.3. PACα and PACβ Lead to an Impairment of Gravitaxis

**Figure 4:**
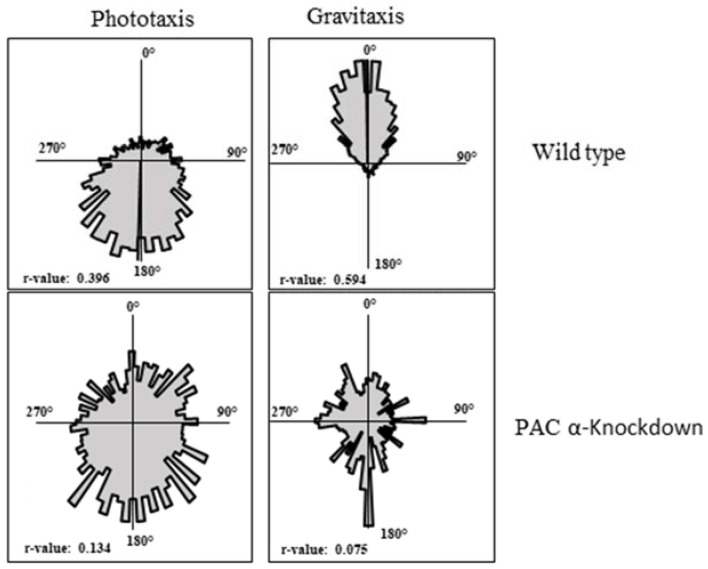

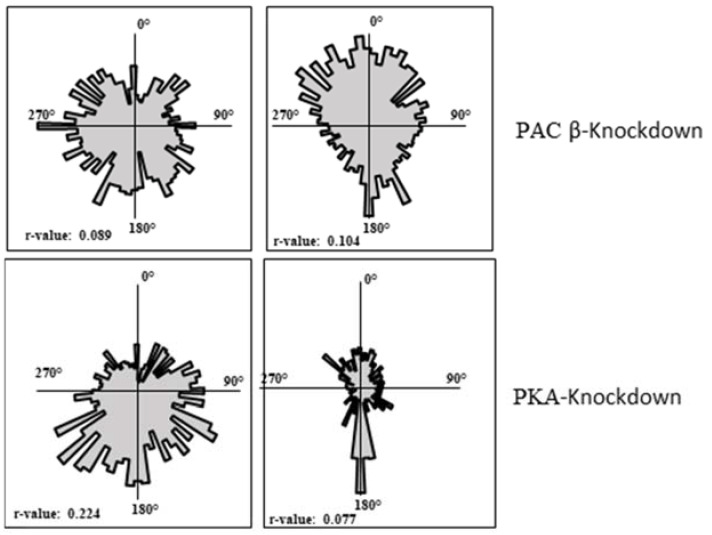
Representative circular histograms showing the directional movement of wild-type, PACα-, PACβ-, and PKA-silenced cells after 4 days of electroporation. Gravitaxis was impaired in PACα- and PACβ-silenced cells.

PKA and PAC are involved in phototaxis and regulate each other’s expression levels. Given that PKA is involved in gravitaxis, it was plausible to assume the involvement of PAC in gravitaxis. In this context, the gravitatic behavior of PACα-, PACβ-, and PKA-silenced cells was analyzed. It was observed that both PACα- and PACβ-silenced cells showed an impaired gravitaxis (Figure 4). Similarly, PACα-, PACβ- and PKA-silenced cells showed an impaired phototaxis (Figure 4). These findings strongly suggest that PKA and PAC share a functional relationship.

## 3. Discussion

Here, we reported the subcellular localization of the catalytic domain of PKA, which was characterized to regulate the phototaxis and gravitaxis of *E. gracilis* [21]. The PKA signal was more abundant in the cell body fraction than the flagella in the Western blot assay, whereas the IIFA further clarified the precise localization of PKA in the reservoir region at the anterior end of the cell. The motor apparatus of *E. gracilis* resides in its anterior region and consists of flagella and PFB that harbors PAC. Therefore, the presence of the PKA in this region is of vital importance. In vitro studies showed that the purified PAC and the isolated PFB fractions of *E. gracilis* carry out the cyclase activity [17]. Similarly, the activation of cAMP-dependent PKA was demonstrated by the heterologously expressed PAC of *E. gracilis* in the oocytes of *Xenopus laevis*, *Drosophila melanogaster*, and the HEK293 cell line [32]. The involvement of PKA in phototaxis and gravitaxis and its spatial proximity to PAC led us to suspect a putative relationship between them.

Interestingly, our results elucidated the connection between PKA and PAC as they regulate each other’s expression levels. However, the nature of the mechanism through which they regulate their expression is not yet precise. In Trypanosoma, protein-coding genes are reported to be transcribed in a polycistronic fashion, as they are tandemly arranged in a polygenic manner in the genome [33]. Therefore, a similar mechanism for PKA and PAC can be assumed in *E. gracilis,* since it is phylogenetically close to Trypanosoma [9,10]. The polycistronic expression of noncoding small nucleolar RNA was reported, but not on the protein-coding genes of *E. gracilis* [34]. Nevertheless, it is still unclear whether polycistronic gene expression is a common scheme in *E. gracilis*. Thus, it can be assumed that PACα and PACβ exist as polycistrons because the silencing of any PAC subunit resulted in the downregulation of the other. However, it is unlikely that PKA, PACα, and PACβ occur as polycistrons, as the PKA expression level remained unaffected in PACβ-silenced cells, whereas the PACα and PACβ expression levels were found to be lower in PKA-silenced cells. The members of a molecular pathway regulate each other through a transcriptional feedback loop [35,36,37]. Since PKA and PAC are involved in the same molecular pathway, it is plausible that a transcriptional feedback loop exists between them.

The impairment of gravitaxis in the PACα-silenced cells can be explained as a synergistic effect of PACα, PACβ, and PKA downregulation, whereas the impairment of gravitaxis in PACβ-silenced cells was due to the reduction in the PAC content, since PACα was also downregulated in PACβ-silenced cells. Collectively, these findings suggested that the silencing of PACα and PACβ resulted in a decrease in the cyclase activity of the PAC, which hampered the activation of PKA. Even though the cyclase activity of PAC is blue light-dependent, it was shown that both heterologously expressed and native PAC exhibit a significant cyclase activity under dark conditions as well [17,32]. Therefore, it seems plausible that by regulating the activity of PKA, PAC also plays a role in gravitaxis.

cAMP is an important secondary messenger that controls several cellular mechanisms in a range of organisms [38]. Major players that regulate cAMP are adenylyl cyclases (ACs) and PDA. PKA is an effector molecule of cAMP, which carries out several cellular tasks through the post-translational modifications of targeted phosphoproteins [39]. PKA is a tetrameric protein that consists of a regulatory (R) dimer subunit and two catalytic (C) subunits [39]. Following the binding of cAMP with the regulatory dimer, the catalytic subunits of PKA are released to phosphorylate the targeted serine and threonine-containing domains of phosphoproteins. The specificity of protein phosphorylation requires the spatial–temporal maintenance of cAMP and the molecules responsible (PKA, PDA, and ACs) for the phosphorylation of target proteins. The task is carried out by a kinase anchoring protein (AKAP) by confining the PKA holoenzyme and PDA in a specific cellular region [40,41]. Therefore, the presence of PKA at the anterior region in close proximity to PAC indicates the involvement of AKAP. AKAP has been suggested to regulate cAMP-mediated signaling by keeping all the cAMP regulatory players to a certain intracellular niche [42]. In this regard, the presence of PKA at the hub of flagellar motor machinery strengthens its role as an essential regulator of flagellar motor proteins. So far, only one characterized flagellar protein, EgPCDUF4201, has been shown to be involved in gravitaxis [23]. It is plausible that PKA regulates EgPCDUF4201, but this is yet to be determined. Moreover, a recent study showed several flagellar proteins that could be considered for further studies for their potential role in light- and gravity-sensing mechanisms in *E. gracilis* regulated by PKA [43]. Notably, Hammond et al. [43] reported several members of the gravitatic and phototactic chains, including the TRP channel in the cell body fraction and EgPCDUF4201 and PKA in the flagella fraction of *E. gracilis* [21,23,24]. However, this study showed the precise localization of PKA at the base of flagella.

Taken together, the subcellular localization of PKA in this study strengthened the proposed working model of gravitaxis and phototaxis in *E. gracilis*. Moreover, the association of PKA and PAC strongly indicated that PKA is a specific downstream regulator of the PAC-mediated cAMP pathway. Thus, it was evident that a molecular cross-talk occurred between light- and gravity-sensing mechanisms in *E. gracilis*. Eventually, these findings gave rise to new questions, such as what are the target proteins of PKA in flagella, and opened new horizons for fundamental research to explore the motor machinery that controls the beating pattern of cilia/flagella.

## 4. Materials and Methods

### 4.1. Cell Culture and Growth Conditions

*Euglena gracilis* KLEBS strain Z was obtained from the algal culture collection center at the University of Göttingen, Germany [44]. Experiments were performed with axenic cells grown in an organic medium [45]. The cultures were grown under continuous light (cool white light intensity of 54 μmol m^−2^ s^−1^) at 20 °C.

### 4.2. Motion Analysis

The motion analysis was performed using a cell tracking software, Wintrack 2000 [46], coupled with a custom-made device (Manual Ecotox), which consisted of a microscope with a horizontal beam path and a CCD camera to observe cell movement for the direction of the gravity vector. Blue light LED provided the phototactic stimulus. The infrared LED (λ = 875 nm) allowed observation without influencing the cells. Cells were transferred to a custom-made disposable glass cuvette, which was positioned vertically in the horizontal microscope to determine the gravitactic orientation. The Wintrack 2000 software was used to analyze the incoming video images, determine the movement vectors and various physiological (cell motility, cell shape, velocity, area of the objects, etc.) and statistical parameters of the swimming cells simultaneously. The orientation precision (r-value) was determined as described previously [46]. The r-value ranged between 0 (when all cells swam randomly) and 1 (when all cells swam in a single direction). Cell movement was represented as a circular histogram with angular sectors [47]. The size of the angular sectors reflected the number of cells moving in a corresponding direction.

### 4.3. dsRNA-Mediated Gene Silencing and Expression Analysis

An RNAi approach was adopted for gene silencing described earlier [23]. Total RNA was extracted using the Trizol reagent (Thermo Fisher Scientific, Waltham, MA, USA) [48], and its concentration was estimated spectrophotometrically using a nano-drop photometer (Thermo Fisher Scientific, USA). The total RNA was transcribed into cDNA using the QuantiTECTreverse transcription kit (QIAGEN, Hilden, Germany). PCR was performed with primers listed in Table S1 to amplify the target gene sequence. The dsRNA was generated and the electroporation of wild-type *E. gracilis* was carried out as described earlier [23,49]. The quantification of the mRNA levels in wild-type gene-silenced *E. gracilis* cells was performed on the CFX96 TouchTM Real-Time PCR detection system using the QuantiTect SYBR Green PCR Kit (QIAGEN, Hilden, Germany) [23]. The cDNA was diluted 1:3 or 1:500 with deionized water before PCR and the gene-specific primers (Table S1) were used for amplification, whereas actin was used as a reference gene.

### 4.4. Biochemistry

#### 4.4.1. Antibodies

The production of custom antibodies against PKA (protein id: ACH72986.1) was outsourced to a commercial firm, SDIX, Newark, Delaware, USA. The antibody was raised using a genomic antibody approach against 91 amino acids (58-148) of PKA [27]. Antirabbit tubulin alpha chain antibody (Catalogue#AS10 680, Agrisera, Vännäs, Sweden) was also used. The commercially available Alexa Fluor^®^ 488-Anti-Rabbit IgG (H + L) (Jackson Immune Research Laboratories, USA), Anti-Mouse IgG-peroxidase antibody (Sigma Aldrich, St. Louis, MO, USA), and anti-Rabbit IgG-Peroxidase antibody (Sigma Aldrich, USA) were used as secondary antibodies in this study.

#### 4.4.2. Protein Sample Preparation from E. gracilis

The cells were harvested by centrifugation (11,000× *g*, 10 min) and washed twice with deionized water. The cell pellet was suspended in lysis buffer (40 mM Tris-HCl, pH 8) supplemented with a protease and phosphatase inhibitor cocktail (10 μL mL^−1^, product number 78,443, Thermo Fisher Scientific, USA). The cell disruption was performed with an ultrasonic homogenizer (Bandelin ultrasonic converter, Sonoplus hd UW 2070 and HF generator, GM 2070, Berlin, Germany) [23]. The samples were processed at 4 °C. The resultant crude lysate was resuspended in acetone (100% *v/v*) and the protein precipitation was performed at −20 °C overnight. The protein fraction was collected by centrifugation at 24,000× *g* and 4 °C for 1 h and then washed twice with 80% acetone. The air-dried protein pellet was suspended in 100 μL of resuspension buffer (7 M urea, 2 M thiourea, 2% CHAPS, and 40 mM DTT) and, subsequently, transferred to a 1.5 mL microcentrifuge tube. Protein solubilization was performed at 30 °C and 1400 cycles min^−1^ for 1 h using a heating shaker (Thermomixer comfort, Eppendorf, USA) and the solubilized protein fraction was collected by centrifugation at 20,000× *g* and room temperature for 5 min. The concentration of protein was determined by the Bradford assay [40].

#### 4.4.3. SDS PAGE and Immunoblotting

The SDS-PAGE was performed as described previously [50]. The separation of protein samples was performed on a 12% SDS polyacrylamide gel, and were stained with Coomassie brilliant blue R250 solution for analysis [51]. The Western blot was performed as described elsewhere [23].

#### 4.4.4. Organelle Fractionation

The separation of flagella and cell body was performed using the method described elsewhere using 15 L of late logarithmic cell culture [29].

#### 4.4.5. Indirect-Immunofluorescence Confocal Microscopy

For indirect-immunofluorescence assay (IIFA), samples were prepared as described earlier [28], and cells were analyzed using fluorophore (Alexa 488) conjugated secondary antibodies with a confocal microscope (SP2 Leica, Wetzlar, Germany).

## Data Availability

Not applicable.

## Supplementary Materials

The following supporting information can be downloaded at: https://www.mdpi.com/article/10.3390/ijms23052776/s1.
