# Supplementary material for: Molecular Cross-Talk between Gravity- and Light-Sensing Mechanisms in Euglena gracilis
Source: Int J Mol Sci. 2022 Mar 3;23(5):2776. doi: 10.3390/ijms23052776 (PMC8911436; doi:10.3390/ijms23052776)
Supplement: Supplementary file 1 [file ijms-23-02776-s001.zip › ijms-1559691-supplementary.pdf]

Supplementary data

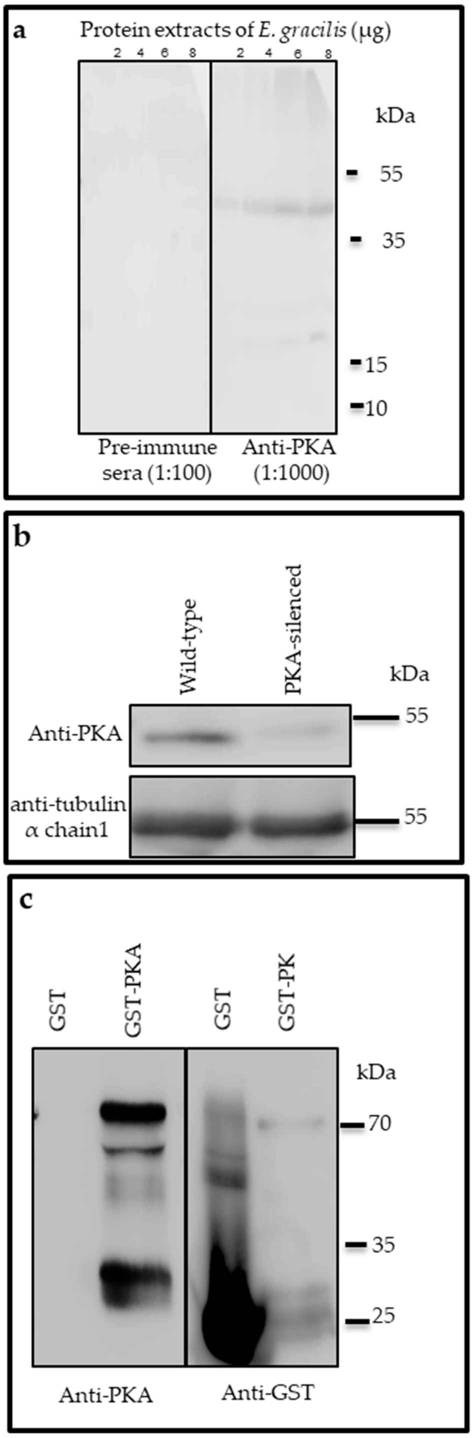

Figure S1. Anti-PKA antibody characterization and validation of specificity. (**a**) Western blot with different concentrations of protein extract. **(b)** Western blot with the protein fraction of wild-type cells and the PKA-silenced cells. **(c)** Western blot of the purified GST-PKA fusion protein.

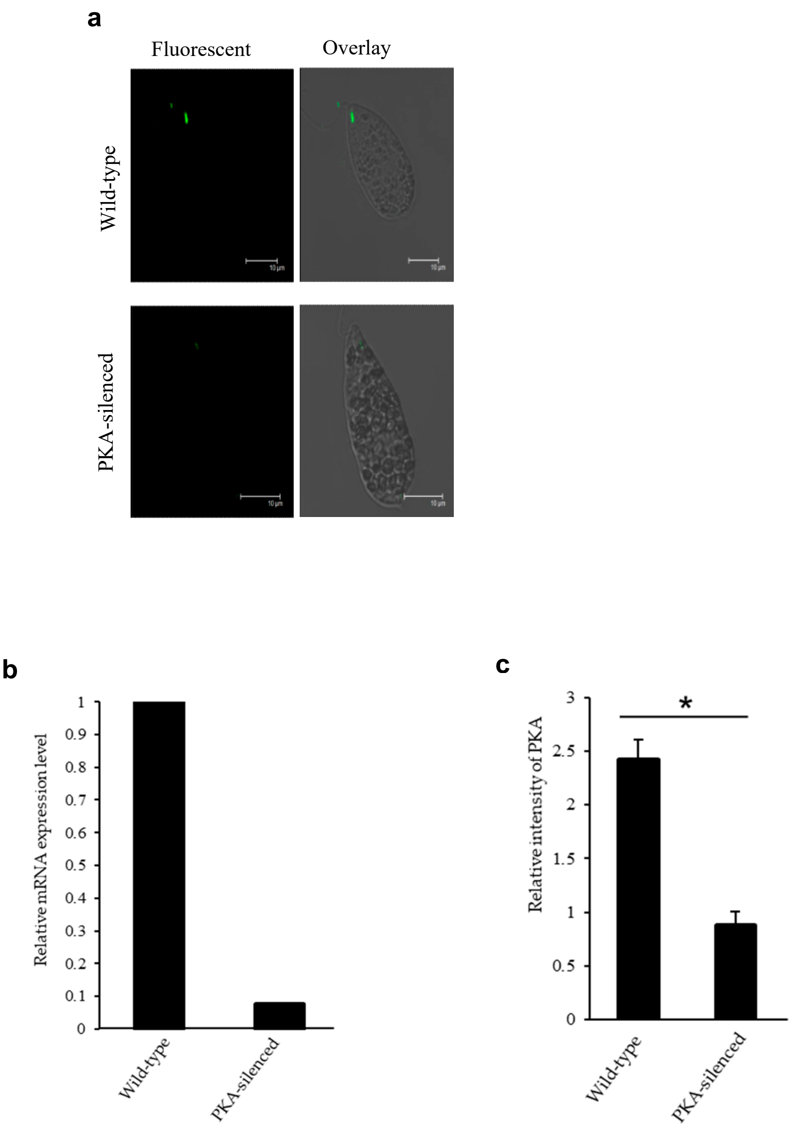

Figure S2. Confirmation of the PKA localization. **(a)** IIFA with Anti-PKA antibody. **(b)** Expression level of PKA normalized to actin. **(c)** Signal intensity in wild-type and PKA-silenced cells. N=50. * represents a significant difference in the student test.

Table S1. Nucleotide sequences (5-3′) of the primers

Name	Sequence
**C-PACα-For**	ATGCTCAATATGATCGACAATTTCGTGGAT
**C-PACα-Rev**	ATGCGGGTGCCGTTCAGAAGGAT
**C-PACβ-Rev**	AATCCTCCTTGACAGAGGTTTGGATGATCT
**C-PACβ-Rev**	CGCTTCTCGGAGCTGGCGGCAGAAAG
**PKA-For**	CACTCTTCGATTTCAATCCAGATGAGATGA
**PKA-Rev**	GAGGAGCTGATTTGCAAGACCACAGA
